# Conversion to mammalian target of rapamycin inhibitors in kidney transplant recipients with de novo cancers

**DOI:** 10.18632/oncotarget.14908

**Published:** 2017-01-30

**Authors:** Chi Yuen Cheung, Maggie Kam Man Ma, Wai Leung Chak, Ka Foon Chau, Sydney Chi Wai Tang

**Affiliations:** ^1^ Department of Medicine, Renal Unit, Queen Elizabeth Hospital, Yau Ma Tei, Hong Kong SAR; ^2^ Department of Medicine, Division of Nephrology, The University of Hong Kong, Queen Mary Hospital, Yau Ma Tei, Hong Kong SAR

**Keywords:** cancer, everolimus, kidney transplant, sirolimus

## Abstract

**Objective:**

To investigate the impact of mammalian target of rapamycin (mTOR) inhibitor conversion together with minimization of calcineurin inhibitor on allograft outcome and patient survival in kidney transplant recipients with post-transplant cancers.

**Methods:**

A retrospective study of all kidney transplant recipients diagnosed to have post-transplant cancers between the period 1/1/1994 and 30/6/2015. Patients were divided into 2 groups: mTOR inhibitor group and non-conversion group. Outcome included allograft function, patient survival, graft survival, acute rejection and cancer recurrence.

**Results:**

115 patients (56 in mTOR inhibitor group and 59 in non-conversion group) were analyzed. Median follow up was 28 months (range: 1 month – 20 years). The allograft function at 1-year remained similar between both groups. There was no significant difference in the patient survival, graft survival and rejection free survival between both groups. More patients in the non-conversion group developed recurrence of cancers than mTOR inhibitor group but statistically not significant.

**Conclusions:**

Use of mTOR inhibitors together with calcineurin inhibitor minimization offer a reasonable option in kidney transplant recipients who developed post-transplant cancers in view of stable renal function, low rejection rate and low cancer recurrence rate.

## INTRODUCTION

Cancer is an important comorbidity in kidney transplantation because the incidence and prevalence of post-transplant cancers have increased in the past 10-15 years [1–3]. Long-term immunosuppression is an important contributor to the increased number of post-transplant cancers. However, it is still not clear how the immunosuppressive regimen should be adjusted for those patients who developed cancers. Although reduction of immunosuppressive agents is widely accepted, there has been concern that it may adversely affect the patient and allograft survival [4–6].

The role of mammalian target of rapamycin (mTOR) inhibitors in kidney transplantation has attracted much attention because of its dual immunosuppressive and antitumor effects [7–10]. However, the role of mTOR inhibitors in patients who developed post-transplant cancers is less certain. One commonly used strategy is to stop the calcineurin inhibitors (CNIs), introduce mTOR inhibitors and then minimize the mycophenolate [11]. Switching from CNIs to mTOR inhibitors has been shown to be associated with regression of post-transplant Kaposi sarcoma [12, 13]. In addition, randomization studies also showed that conversion from CNIs to sirolimus reduced the risk of non-melanoma skin cancer (NMSC) recurrence after kidney transplantation [8, 9]. However, complete elimination of CNIs may be associated with a greater risk of acute or chronic rejection and graft failure. On the contrary, minimization of CNI doses in association with mTOR inhibitors seems to be well tolerated without increasing the risk of rejection [14].

Although there have been some case series concerning the use of mTOR inhibitors in kidney transplant recipients after cancer diagnosis, the main approach in these studies was the elimination of the CNIs. On the other hand, information on the use of mTOR inhibitors with CNI minimization in post-transplant cancers was scarce. Hence we investigate the impact of conversion to mTOR inhibitors together with CNI minimization on the allograft outcome and patient survival in kidney transplant recipients who developed de novo cancers.

## RESULTS

Total 1227 kidney transplant recipients had follow up during the study period and 124 (10.1%) of them developed cancers. Among these cancers, 23 were hematological cancers (all were post-transplant lymphoproliferative disorders (PTLD)) and 101 were solid organ cancers. The most common sites of solid organ cancers included kidney (*n* = 19), colorectum (*n* = 13), liver (*n* = 11), lung (*n* = 10) and breast (*n* = 6). The mean age at transplant was 44.5 +/- 12.1 years and the mean age at diagnosis of cancer was 53.8 +/- 12.1 years. The median duration from transplant to cancer was 8.8 years (2 months - 26.8 years). The overall mortality was 59.7 (74/124) %. The most common causes of death were cancer progression (*n* = 37), followed by sepsis (*n* = 21) and ischemic heart disease (*n* = 6). On the other hand, 19 patients had graft failure (14 due to chronic allograft nephropathy, 1 due to acute rejection and 4 due to unknown causes).

In order to study the effects of mTOR inhibitors in our cohort, 9 patients were excluded from analysis. Seven were on mTOR inhibitors before cancer and 2 had graft nephrectomy (one due to renal cell carcinoma and the other due to non-Hodgkin lymphoma within the grafts) with subsequent withdrawal of immunosuppression. As a result, 115 patients were further analyzed (Table 1). The median follow up was 28 months (range: 1 month - 20 years). Fifty-six patients belonged to the mTOR inhibitor group (mean follow up 40 +/- 39 months) and 59 belonged to the non-conversion group (mean follow up 50 +/- 59 months). There was no significant difference in the follow-up duration between both groups (*P* = 0.26). Their baseline demographic and clinical characteristics were depicted in Table 2.

**Table 1:** Number of patients according to the site and stage of cancer

Sites of cancer (*n*= 115)	Localized (*n*= 64)	Advanced (*n*= 51)
PTLD	5	13
Kidney and ureter	17	3
Bladder	4	0
Prostate	3	1
Testis	1	1
Cervix	3	0
Ovary	1	1
Vulva	1	0
Esophagus	1	1
Stomach	2	2
Colorectum and anus	4	9
Liver	7	4
Pancreas	0	1
Breast	1	4
Lung	3	7
Nasopharynx	0	1
Oral cavity and tongue	1	1
Thyroid	3	0
NMSC	4	0
Melanoma	1	0
KS	2	2

KS: Kaposi sarcoma; NMSC: non melanoma skin cancer; PTLD: post-transplant lymphoproliferative disorders;

Definition of localized and advanced disease shown in the Material and Methods section.

**Table 2:** Demographic and clinical characteristics of patients with and without conversion to mTOR inhibitor-based therapy

	mTOR inhibitor (*n*= 56)	Non-conversion (*n*= 59)	*P* value
Age at cancer (years)	54.6 +/- 10.7	53.6 +/- 12.7	0.64
Age at transplant (years)	44.6 +/- 11.8	44.8 +/- 12.3	0.94
Duration from transplant to cancer (years)	9.9 +/- 6.5	8.9 +/- 7.2	0.39
Male, n (%)	36 (64.3)	31 (52.5)	0.20
Causes of ESRD, n (%) Glomerulonephritis Diabetes Hypertension Unknown Others	27 (48.2) 5 (8.9) 6 (10.7) 6 (10.7) 12 (21.5)	29 (49.2) 2 (3.4) 3 (5.1) 10 (16.9) 15 (25.4)	0.46
Deceased / Living transplant, n (%)	45 (80.4) / 11 (19.6)	52 (88.1) / 7 (11.9)	0.25
First / Second transplant, n (%)	53 (94.6) / 3 (5.4)	58 (98.3) / 1 (1.7)	0.28
Biopsy proven rejection before cancers, n (%)	15 (26.8)	17 (29.8)	0.81
Localized / Advanced, n (%)	32 (57.1) / 24 (42.9)	32 (54.2) / 27 (45.8)	0.74
Hematological / Solid organ cancers, n (%)	11 (19.6) / 45 (80.4)	7 (11.9) / 52 (88.1)	0.25
Induction therapy, n (%)	7 (12.5)	13 (22.0)	0.18
CNI therapy, n (%) Cyclosporine Tacrolimus	36 (64.3) 20 (35.7)	43 (72.9) 16 (27.1)	0.32 0.32
Concomitant immunosuppression, n (%) Prednisolone Azathioprine Mycophenolate	55 (98.2) 23 (41.1). 19 (33.9)	56 (94.9) 28 (47.5) 6 (10.2)	0.33 0.49 <0.01
Cyclosporine trough level (ng/mL)	126 +/- 44	126 +/- 25	0.99
Tacrolimus trough level (ng/mL)	6.3 +/- 1.3	6.3 +/- 1.4	0.99
Median serum creatinine (umol/L)	114 (68-271)	104 (66-261)	0.71
eGFR (ml/min/1.73m2)	58 +/- 19	57 +/- 19	0.84
Amount of proteinuria (g/day)	0.18 +/- 0.19	0.28 +/- 0.33	0.20

CNI: calcineurin inhibitor; ESRD: end stage renal disease; eGFR: estimated glomerular filtration rate

Table 3 showed the immunosuppressive regimen before and after diagnosis of cancers in both groups. In the mTOR inhibitor group, azathioprine was withdrawn in 23 patients while mycophenolate was withdrawn in 19 patients. On the other hand, only 3 patients had elimination of CNIs. They were then replaced immediately with mTOR inhibitors (sirolimus in 35 patients and everolimus in 21 patients). As a result, 53 patients (94.6%) were on prednisolone + CNI + mTOR inhibitor regimen and 3 (5.4%) patients were on prednisolone + mTOR inhibitors. The dose of CNIs was substantially reduced after the diagnosis of cancer. On the other hand, azathioprine was withdrawn in 9 patients and mycophenolate in 3 patients in the non-conversion group. Although a smaller dose of CNIs was also used in the non-conversion group, the dose reduction of CNIs was not as much as that in the mTOR inhibitor group. As a result, the trough levels of both cyclosporine and tacrolimus were significantly lower in the mTOR inhibitor group (Table 4). The trough level of sirolimus was 8.2 +/- 2.1 ng/mL while the trough level of everolimus was 4.9 +/- 1.2 ng/mL.

**Table 3:** Change of immunosuppressive regimen before and after cancer

	mTOR inhibitor (*n* = 56)	Non-conversion (*n*= 59)
Drug regimen before cancer, *n* (%) Pred/Aza/CsA Pred/Aza/FK Pred/MMF/CsA Pred/MMF/FK Pred/CsA Pred/FK CsA/Aza	16 (28.6) 6 (10.7) 8 (14.3) 11 (19.6) 11 (19.6) 3 (5.4) 1 (1.8)	19 (32.2) 6 (10.2) 1 (1.7) 5 (8.5) 20 (33.9) 5 (8.5) 3 (5.0)
Dosage before cancer (mg) Pred Aza MMF CsA FK	7.5 (5.0-7.5) 50 (25-100) 1000 (500-1500) 150 (50-250) 3.0 (1.0-5.0)	7.5 (5.0-30) 50 (25-100) 1000 (1000-1500) 175 (100-400) 4.0 (2.0-14)
Drug regimen after cancer, *n* (%) Pred/Aza/CsA Pred/Aza/FK Pred/MMF/CsA Pred/MMF/FK Pred/CsA Pred/FK CsA/Aza CsA Pred/CsA/Sirolimus Pred/FK/Sirolimus Pred/Sirolimus Pred/CsA/Everolimus Pred/FK/Everolimus Pred/Everolimus	0 (0) 0 (0) 0 (0) 0 (0) 0 (0) 0 (0) 0 (0) 0 (0) 23 (41.1) 11 (19.6) 1 (1.8) 10 (17.8) 9 (16.1) 2 (3.6)	14 (23.7) 3 (5.1) 0 (0) 3 (5.1) 26 (44.1) 11 (18.6) 1 (1.7) 1 (1.7) 0 (0) 0 (0) 0 (0) 0 (0) 0 (0) 0 (0)
Dosage after cancer (mg) Pred Aza MMF CsA FK	5.0 (5.0-7.5) 0 (0) 0 (0) 50 (0-175) 1.5 (0-4.0)	5.0 (0-10) 50 (0-100) 1000 (0-1000) 150 (100-300) 3.5 (0-4.5)

Aza: azathioprine; CsA: cyclosporine; FK: tacrolimus; MMF: mycophenolate mofetil; Pred: prednisolone;

**Table 4:** Renal function and amount of proteinuria after cancers

	mTOR inhibitor (*n*= 56)	Non-conversion (*n*= 59)	*P* value
Median serum creatinine (umol/L) 6-month 1-year	113 (62-321) 119 (45-815)	107 (57-334) 106 (66-355)	0.71 0.32
eGFR (ml/min/1.73m^2^) 6-month 1-year	59 +/- 21 58 +/- 29	59 +/- 22 54 +/- 20	0.99 0.50
Proteinuria, 1-year (g/day)	0.68 +/- 0.92	0.38 +/- 0.56	0.17
CsA trough level (ng/mL)	52 +/- 17	75 +/- 23	<0.01
FK trough level (ng/mL)	3.6 +/- 1.0	4.6 +/- 1.3	0.04

CsA: cyclosporine; eGFR: estimated glomerular filtration rate; FK: tacrolimus

**Figure 1:**
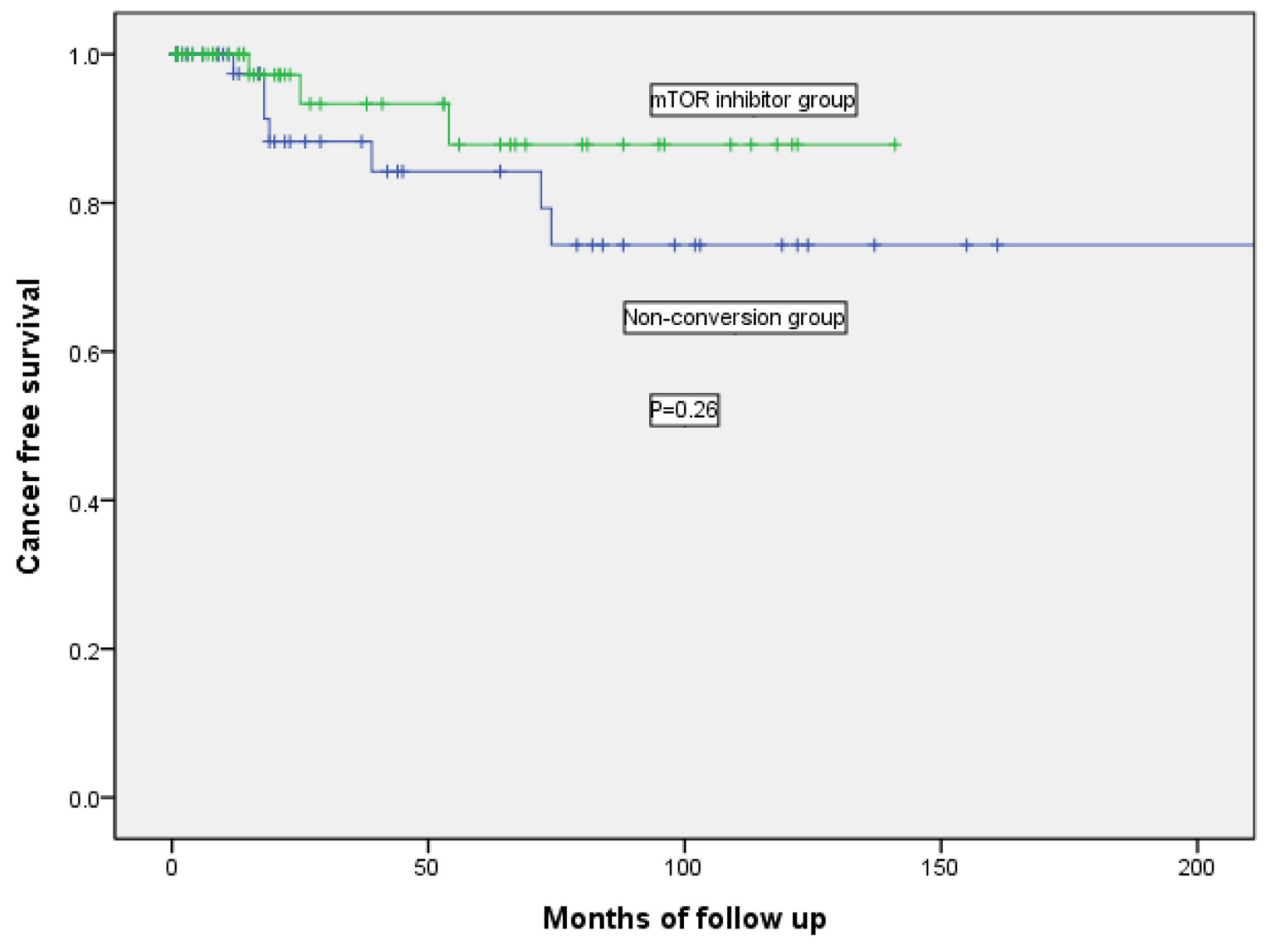
Kaplan-Meier curve showing the cancer free survival in mTOR inhibitor group and non-conversion group

Renal function remained similar between both groups before cancer development and 1 year after cancer (Table 4). If only those patients with eGFR > 30 ml/min/1.73m^2^ were analyzed, the eGFR at 1 year was better in mTOR inhibitor group (*n* = 41) than non-conversion group (*n* = 27) although it was not statistically significant (61 vs 58 ml/min/1.73m^2^, *P* = 0.70). Only 4 patients in our cohort developed biopsy proven acute rejection after cancer (2 in each group). Two had type 1A acute cellular rejection, 1 had acute antibody-mediated rejection and 1 had borderline acute rejection. There was no significant difference in the rejection free survival between both groups (*P* = 0.48). More patients (7/59, 11.9%) in the non-conversion group developed recurrence of cancers than mTOR inhibitor group (3/56, 5.4%). However, there was no significant difference in the disease free survival (*P* = 0.26, Figure 1).

Total 71 patients (28 in mTOR inhibitor group and 43 in non-conversion group) died during the follow up period. Twelve patients in the mTOR inhibitor group and 24 in the non-conversion group died of cancer progression. In the mTOR inhibitor group, all patients who died of cancer already had advanced disease during diagnosis. Five patients died of carcinoma of lung, 2 carcinoma of colon, 1 carcinoma of esophagus, 1 carcinoma of breast, 1 renal cell carcinoma, 1 nasopharyngeal carcinoma and 1 carcinoma of ovary. On the other hand, 22 patients who died in the non-conversion group had advanced cancers (5 PTLD, 4 colon, 4 liver, 2 stomach, 2 lung, 1 breast, 1 prostate, 1 pancreas, 1 kaposi sarcoma and 1 oral cavity) while 2 patients had cancer recurrence (1 liver and 1 esophagus). The 1-year and 3-year patient survival in mTOR inhibitor group were 80.4% and 52.0% respectively while the 1-year and 3-year patient survival in non-conversion group were 83.0% and 44.7% respectively (*P* = 0.17). On the other hand, 5 patients had graft failure (2 due to chronic allograft nephropathy and 3 due to unknown causes) in the mTOR inhibitor group and 11 patients lost their grafts (1 due to acute antibody-mediated rejection and 10 had chronic allograft nephropathy) in the non-conversion group. For the 2 patients who had chronic allograft nephropathy in the mTOR inhibitor group, 1 patient already had eGFR less than 30ml/min/1.73m^2^ during conversion while the other patient had graft failure 5 years after conversion to mTOR inihibitor. The 1-year and 3-year death-censored graft survival in mTOR inhibitor group were 97.9 % and 90.3% respectively while the 1-year and 3-year death-censored graft survival in non-conversion group were 93.2% and 80.2% respectively (*P* = 0.17).

There was no significant difference in the distribution of hematological malignancies and solid organ cancers in both treatment arms (Table 2). If we just focused on those patients with hematological malignancies (all PTLD in our cohort), there were 4 with localized disease and 7 with advanced disease in the mTOR inhibitor group while there were 1 with localized disease and 6 with advanced disease in the non-conversion group. There was no significant difference in the localized or advanced PTLD in both groups (*P* = 0.60). None of the patients with PTLD developed cancer recurrence or acute allograft rejection. Eight patients died (7 due to sepsis and 1 due to ischemic heart disease) in the mTOR inhibitor group and 7 patients died (5 due to cancer progression and 1 due to sepsis) in the non-conversion group. There was no significant difference in patient survival (*P* = 0.12) or death-censored graft survival (*P* = 0.44) between both treatment groups. For solid organ tumors, there was also no significant difference in patient survival (*P* = 0.16), death-censored graft survival (*P* = 0.19) and cancer recurrence free survival (*P* = 0.31) between both groups of patients.

Five patients discontinued mTOR inhibitors within the first 2 years of study period because of various side effects, 1 due to severe aphthous and colonic ulcers, 1 due to hemolytic uremic syndrome, 1 due to severe gastrointestinal upset, 1 due to interstitial lung disease and `1 due to increasing proteinuria. On the other hand, none of the patients in the non-conversion group reported serious side effects.

## DISCUSSION

There has been emerging data showing that mTOR pathway plays a significant role in the development of different types of cancers [11]. Although the protective effect of mTOR inhibitors on cancer development in transplant recipients has been shown in different studies [10, 12, 15, 16], a recent meta-analysis study by Yanik EL et al. showed that there was no association between sirolimus and the incidence of cancers after excluding NMSC [17]. Currently it is still not certain what would be the best immunosuppressive drug regimen in the kidney transplant recipients who developed de novo cancers. Different studies have demonstrated that mTOR inhibitors might be beneficial in the treatment of Kaposi sarcoma, recurrent skin cancer, PTLD and renal cell carcinoma [18]. The most common practice is to withdraw CNIs abruptly and then replace with mTOR inhibitors [6, 11]. However, complete avoidance or withdrawal of CNIs could be associated with a greater risk of acute or chronic rejection and graft failure [14, 19]. In addition, a retrospective study showed that conversion to mTOR inhibitors after CNI withdrawal might increase the risk of developing class II donor-specific antibodies, which probably favor chronic antibody-mediated rejection and thus reduce allograft survival [20].

Unlike other studies [6, 21, 22], only 3 patients (5.4%) in the mTOR inhibitor group in our study had CNI withdrawal. Approximately 95% of patients were switched to the combination of prednisolone, low dose CNI and mTOR inhibitor. In our cohort, the trough level of sirolimus was 8.2 +/- 2.1 ng/mL while the trough level of everolimus was 4.9 +/- 1.2 ng/mL. On the other hand, the CNI trough level was maintained at a much lower level when compared with the patients in the non-conversion group. In the mTOR inhibitor group, the cyclosporine trough level was 52 +/- 17 ng/mL and the tacrolimus trough level was 3.6 +/- 1.0 ng/mL. Despite significant reduction of CNI dosage after diagnosis of cancer, the overall rejection rate remained low in our study. Only 4 patients (3.5%) suffered from acute rejection (2 from each group) after a median follow up of 28 months and there was no significant difference in the rejection free survival between both treatment arms. Although there were fewer episodes of cancer recurrence (5.4% vs 11.9%) in the mTOR inhibitor group than in the non-conversion group, the difference was not statistically significant. One possible explanation was that the follow-up period in our cohort was too short to show the effectiveness of mTOR inhibitors in the prevention of cancer recurrence.

The eGFR of patients in the mTOR inhibitor group at both 6-month and 1-year was similar to those in the non-conversion group. The lack of significant improvement in renal function in the mTOR inhibitor group could be the result of the relatively late mTOR inhibitor conversion in our cohort (mean 10.4 years). In addition, another study also showed that eGFR improvement was less among patients who had mTOR inhibitor conversion due to cancers when compared with those who had conversion due to chronic graft dysfunction [23].

In the mTOR inhibitor group, all patients who died of cancer progression were due to advanced-stage solid organ tumors. On the other hand, none of the patients with PTLD (*n* = 11) died of malignancies. This is in contrast to the patients with PTLD in the non-conversion group (*n* = 7) where 5 of them died of cancer progression. It is not clear whether this discrepancy could be due to the potent anti-proliferative effect of mTOR inhibitors. However, it seems that mTOR inhibitors were less effective in patients with advanced stage or disseminated solid organ cancers. In addition, the optimal dose of mTOR inhibitors for treatment of various tumors remains unknown and the failure of treatment in some cancers could be the result from the lower dose of mTOR inhibitors used in our cohort.

The major limitations of this study included retrospective design and heterogeneity of different types and stages of cancers which made it difficult to compare our results with other studies. In addition, we could not analyze sirolimus and everolimus individually because of the low overall rate of mTOR inhibitor use. Since the number of patients with post-transplant cancers is relatively small, it is highly unlikely that a prospective, randomized trial with an adequate sample size and duration of follow-up can be performed to detect the differences of using mTOR inhibitor in these patients. As a result, retrospective studies based on institutional experiences, such as our study, are still an attractive option for providing further knowledge about the role of mTOR inhibitor for preventing or treating post-transplant cancers. In fact, our study has already recruited the largest number of kidney transplant recipients who had mTOR inhibitor conversion due to post-transplant cancers. Moreover, the presence of a control group in our study allows comparisons between patients converted to mTOR inhibitors and those remained on CNI-based therapy.

In conclusion, it is still uncertain whether conversion to mTOR inhibitors after cancer development will have any benefit in long-term patient and graft survival in kidney transplant recipients. Our study shows that use of mTOR inhibitors together with CNI minimization may be able to offer a reasonable option in view of the relatively stable renal function, very low rejection rate and low cancer recurrence rate. However, treatment should also be individualized according to the different clinical condition in each patient. Further studies, especially on the optimal dose of mTOR inhibitors in post-transplant solid organ tumors, are required.

## MATERIALS AND METHODS

This was a retrospective study of all kidney transplant recipients who were diagnosed to have post-transplant cancers and had been followed up in 2 large transplant centers in Hong Kong, namely Queen Elizabeth Hospital and Queen Mary Hospital, between the period 1/1/1994 and 30/6/2015. This study was approved by the research ethics committee. All cancer events were collected from the Hong Kong Renal Registry database. The Renal Registry is a direct, online, computerized registry system developed by the Central Renal Committee, Hospital Authority (HA), to collect data of all patients who received renal replacement therapy under HA. The data is entered directly by renal doctors or nurses of the individual renal unit and each unit can access their own patient data [24]. It is therefore an accurate and up-to-date record of all comorbidities and complications of all renal replacement therapy patients including kidney transplant recipients managed under the HA. All cancers were diagnosed and verified with histology and other relevant information, such as radiological imaging, and coded according to the 10th World Health Organization International Classification of Disease (WHO ICD-10). All basic demographic and clinical data were extracted patients’ medical record.

All patients in our study received CNIs, either tacrolimus or cyclosporine, as part of the maintenance immunosuppressive therapy after kidney transplantation.. When cancer was diagnosed, the dose of immunosuppressive drugs was reduced. Starting from 2006, those kidney transplant recipients with newly diagnosed cancers would have their immunosuppressive regimen converted to an mTOR inhibitors (either sirolimus or everolimus)-based therapy which consisted of abrupt withdrawal of mycophenolate / azathioprine together with either CNI minimization or elimination. However, mTOR inhibitors would not be considered in those patients who refused conversion therapy. As a result, the kidney transplant recipients with de novo cancers were divided into 2 groups; namely mTOR inhibitor group (conversion to either sirolimus or everolimus after cancer) and non-conversion group. In mTOR inhibitor group, the initial doses of sirolimus and everolimus were 2 mg per day and 0.75 mg twice daily respectively. The doses of sirolimus and everolimus were then adjusted according to the whole blood trough concentration. The target range was 5-10 ng/mL for sirolimus and 3-8 ng/mL for everolimus. The CNI doses were concurrently reduced to reach our target trough levels of 3-5 ng/mL for tacrolimus and 50-80 ng/mL for cyclosporine. The outcome included the allograft function, patient survival, graft survival, acute rejection and cancer recurrence. Changes in allograft function were determined by comparing serum creatinine level and estimated glomerular filtration rate (eGFR) at 6 month and 1 year after diagnosis of cancers to that at the time of diagnosis. eGFR is calculated by the abbreviated MDRD equation: 186 x (Creatinine / 88.4)-1.154 x (Age)-0.203 x (0.742 if female) x (1.210 if black) [25].

In order to study the effects of mTOR inhibitors in patients with established cancers, patients who already received mTOR inhibitors before cancers were excluded. For solid organ cancers, localized disease was defined as those cancers confined to the primary site of organ while advanced disease was defined for those with lymph node or distant metastasis. For hematological malignancies, localized disease was defined as involving single lymph node region while advanced disease was defined as involving 2 or more lymph node regions or extra-lymphatic organs.

### Statistical analyses

SPSS (SPSS 20.0, Inc., Chicago, IL USA) was used to perform the statistical analyses. Continuous data were either expressed as mean +/- standard deviation (SD) or median (range) and categorical data were expressed as percentages. Categorical data were compared with chi-square or Fishers exact tests while continuous data were compared with t-test.or Mann-Whitney U test. Log rank test and Kaplan Meier survival analysis were used to compare the patient survival, graft survival, rejection free survival and cancer free survival after diagnosis of cancer between the 2 groups. For purposes of the analyses and defining years of follow-up, patients were censored at the time of event, the date of death, the last reported contact or 31st December 2015. A P-value of less than 0.05 was defined as statistically significant.

All authors read and approved the final manuscript
